# The quantification of zebrafish ocular-associated proteins provides hints for sex-biased visual impairments and perception

**DOI:** 10.1016/j.heliyon.2024.e33057

**Published:** 2024-06-13

**Authors:** Hamid Niksirat, Valentina Siino, Christoph Steinbach, Fredrik Levander

**Affiliations:** a Faculty of Fisheries and Protection of Waters, CENAKVA, University of South Bohemia in České Budějovice, Vodňany, Czech Republic; b Department of Immunotechnology, Lund University, Lund, Sweden; c National Bioinformatics Infrastructure Sweden (NBIS), Science for Life Laboratory, Lund University, Lund, Sweden

**Keywords:** Proteomics, Protein, Sexual dimorphism, Eye

## Abstract

Biochemical differences between sexes can also be seen in non-sexual organs and may affect organ functions and susceptibility to diseases. It has been shown that there are sex-biased visual perceptions and impairments. Abundance differences of eye proteins could provide explanations for some of these. Exploration of the ocular proteome was performed to find sex-based protein abundance differences in zebrafish *Danio rerio*. A label-free protein quantification workflow using high-resolution mass spectrometry was employed to find proteins with significant differences between the sexes. In total, 3740 unique master proteins were identified and quantified, and 49 proteins showed significant abundance differences between the eyes of male and female zebrafish. Those proteins belong to lipoproteins, immune system, blood coagulation, antioxidants, iron and heme-binding proteins, ion channels, pumps and exchangers, neuronal and photoreceptor proteins, and the cytoskeleton. An extensive literature review provided clues for the possible links between the sex-biased level of proteins and visual perception and impairments. In conclusion, sexual dimorphism at the protein level was discovered for the first time in the eye of zebrafish and should be accounted for in ophthalmological studies. Data are available via ProteomeXchange with identifier PXD033338.

## 1 Introduction

Sexual reproduction can cause significant differences in the composition of not only sexual organs but many other non-sexual organs of each sex [1,2]. The body of each sex must be physiologically adapted in such a way to be able to support gamete production and parental care [1]. This kind of sexual dimorphism can affect the way each sex deals with metabolites, toxins, and drugs and, eventually their disease susceptibility. For example, several studies showed sex-based differences in the occurrence of cancer, cardiovascular diseases, visual impairment, and so on. Also, sex can affect drug delivery, especially in nanomedicine [3]. Therefore, sex must be considered an important factor during the investigation of disease biomarkers and drug development to yield effective and unbiased results.

Earlier studies revealed the presence of sexual dimorphism in some visual impairments and perception. Blindness and other visual impairments are the main causes of disability that affect millions across the globe each year. However, it has been shown that women are more susceptible to such disorders. For example, long-term studies revealed that the prevalence of cataract [4] and age-related macular degeneration (AMD) are higher among women, especially at older ages [5]. In addition, researchers have found differences in visual perception and cognitive bias between the two sexes that may affect the ability to perform a task in a sex compared to the opposite one [6,7].

The Zebrafish *Danio rerio* is a popular model organism for human developmental, toxicological, and medical research. It has been discovered that 70 % of human genes have at least one orthologue in zebrafish [8]. In addition, up to 82 % of genes responsible for disease in humans possess a zebrafish orthologue. The Zebrafish eye has high similarities in morphology, physiology, and function with the human eye and has long been used as an ocular model for studying human ocular diseases [9,10]. Especially, the study of the regenerative mechanism of the retina in zebrafish is important since it may help development of therapeutic approaches in humans [11].

Developments in high-resolution mass spectrometry and upgraded proteomic workflows and software have resulted in the generation of long lists of precisely identified and quantified proteins for different biological systems. Therefore, in the present study, we applied high-resolution label-free proteomics for identification and quantification of proteins in the eyes of male and female zebrafish. The results presented here include a comprehensive list of proteins found in zebrafish eyes, and further establish an inventory of proteins with sex-dependent levels that may provide clues about sex-based differences in, for instance, visual impairments and perception. Our results may also give support for future developmental, behavioural, toxicological and medical experiments on the zebrafish eye.

## 2 Materials and methods

### 2.1 Animals

Wild-type AB line adult cohort male (n = 6) and female (n = 6) zebrafish *Danio rerio* were used (2 years old). The spinal cords of animals were severed after anesthetizing in ice water, and the eyes were immediately removed and frozen in liquid nitrogen (−196 °C) in a sterile environment. Samples were kept at −80 °C until proteomics processing. All protocols for animal handling were approved by the Institutional Animal Care and Use Committee at University of South Bohemia in České Budějovice.

### 2.2 Protein extraction and in-gel digestion

Extraction of protein from eyes and in-gel digestion were performed as in Niksirat et al. [12] and Niksirat et al. [1]. Briefly, the eye samples were homogenized in 1 % SDS by probe sonication in an ice bath, and supernatants were recovered after centrifugation, followed by protein concentration measurements by the Micro-Lowry method. Protein extracts from each sample (100 μg protein) were individually combined with a sample buffer (62.5 mM Tris-HCl, pH 6.8, 25 % glycerol, 5 % mercaptoethanol, 2 % SDS, 0.01 % bromophenol blue, Sigma-Aldrich, USA) and heated at 95 °C for 5 min. The resulting protein mixtures were separated by electrophoresis approximately 2 cm into 12 % SDS-PAGE using Criterion Precast TGX Gels (BioRad, USA). Gel Code Blue Stain Reagent (Pierce, USA) was applied for gel staining. Subsequently, lanes were divided into smaller sections, subjected to destaining in a solution of 50 % acetonitrile (ACN) and 25 mM NH_4_HCO_3_, and then dried using Speed-Vac (SAVANT, SPD131DDA, Thermo Scientific, USA).

Reduction of gel slices was conducted by treating them with 10 mM DL-dithiothreitol in 100 mM NH_4_HCO_3_ for 1 h at 56 °C. Additionally, alkylation was performed by introducing 55 mM iodoacetamide in 100 mM NH_4_HCO_3_ and incubation for 45 min at room temperature in darkness. Gel pieces underwent two washes with 100 mM NH_4_HCO_3_ and ACN, and the final dried state was achieved using Speed-Vac.

For enzymatic digestion, Trypsin (12.5 ng ml^−1^ modified Trypsin V5111, Promega Biotech AB, Sweden) in 50 mM NH_4_HCO_3_ was added to the dehydrated gel pieces. After storing the samples for 30 min at 4 °C, they underwent an overnight incubation at 37 °C. Peptide extraction was accomplished using 5 % formic acid (FA) and 75 % ACN by incubating the gels for 30 min at room temperature. This process was iterated twice to yield three extractions, and a fourth extraction was performed using ACN alone with a 10-min incubation. Following the combination of extracts, the volume was reduced using Speed-Vac, and the resulting samples were dissolved in 10 μl of 0.1 % FA.

### 2.3 Mass spectrometry

Protein digests were loaded onto an EASY nLC 1200 LC system (Thermo Fisher Scientific, Germany) after resuspension in 0.1 % FA. The analytical column was a fused silica capillary (75 μm × 16 cm Pico Tip Emitter, New Objective, USA) packed in-house with ReproSil-Pur 1.9 μm (Dr. Maisch GmbH, Germany) C18 material, cut to 15 cm length. Peptides were separated using an 80 min method in 0.1 % FA with a 3 min linear gradient from 5 to 10 % solvent B (80 % ACN, 0.1 % FA), followed by a 60 min linear gradient from 10 % to 25 % solvent B, a subsequent 5 min linear gradient from 25% to 40 % solvent B, and finally a 5 min linear gradient from 40 % to 90 % solvent B and finally a 7 min wash at 90 % B at a constant flow rate of 250 nl/min. The LC system was coupled online with a Q Exactive HF-X (Thermo Fisher Scientific, Germany) mass spectrometer employing data-dependent acquisition (DDA) in positive ion mode. Full MS scans were acquired with an automatic gain control (AGC) target value setting of 3 × 10^6^ ions and a maximum injection time of 50 ms and, with 120000 full width half maximum (FWHM) at *m*/*z* 200 MS1 scans between 375 and 1500 *m*/*z*. A top 20 method selecting peptide ions with charge 2 to 6 for higher energy collision-induced dissociation (HCD) fragmentation was used, with a normalized collision energy of 27 for MS/MS and with 40 s dynamic exclusion. MS/MS spectra were acquired at 15000 FWHM resolution with an AGC target of 1 × 10^5^ ions and a maximum injection time of 20 ms and isolation windows of 1.2 *m*/*z*.

### 2.4 Data processing and analysis

Proteowizard version 3.0.11841 was used for the conversion of raw data files to both mzML [13] and MGF (http://www.matrixscience.com) formats. Dinosaur version 1.1.3 [14] was used for detection of MS peptide features, followed by uploading of data files to Proteios Software Environment version 2.20-dev, build 4646 [15] for subsequent processing. MS/MS peptide identification was carried out using MS-GF+ (v2019.06.28) [16] and X!Tandem [17] (Alanine 2017.2.1.4, http://www.thegpm.org/TANDEM/) in parallel, and the search engine results were combined and filtered in Proteios. Precursor tolerance 7 ppm and MS/MS fragment tolerance 0.02 Da were set for the MS/MS searches. The search database was the UniProt Zebrafish proteome version as of November 26, 2018, with reverse protein (decoy) entries appended, yielding a total of 94318 protein entries. Search modifications were: carbamidomethylation of cysteines and variable oxidation of methionine and protein N-terminal acetylation. Identifications passing a PSM (peptide-spectrum-match) false discovery rate (FDR) threshold of 0.002 in any of the samples in the dataset were matched to features in other samples if the MS/MS PSM identification passed FDR<0.1 in the present sample, when matching between runs and propagating feature identities using default settings in Proteios with a previously described label-free quantification workflow [18]. The table with peptide abundances was first normalized with Cyclic Loess normalization in NormalyzerDE (v. 1.6.0) [19] and the normalized (and log2 transformed) output was used for protein roll-up utilizing a re-implementation of RRollup [20] (https://github.com/ComputationalProteomics/ProteinRollup) with a requirement of at least two peptides per protein. No imputation of missing values was performed during data processing and analysis.

The protein-level abundance data were subjected to empirical Bayes *t*-test (Limma) [21] between samples of the two sexes in NormalyzerDE, with Benjamini-Hochberg adjustment of p-values for FDR filtering, with the adjusted p-values referred to as q-values hereafter. In some cases the protein list contained multiple protein groups originating from the same gene. To remove redundant protein entries, the table was sorted on q-values, and the entry with the lowest q-value for each gene was kept. OmicLoupe [22] was also applied for the visualization of normalized data and the creation of volcano and PCA plots. STRING (v11.0) was used to show interactions between proteins [23].

## 3 Results and discussion

**Fig. 1:**
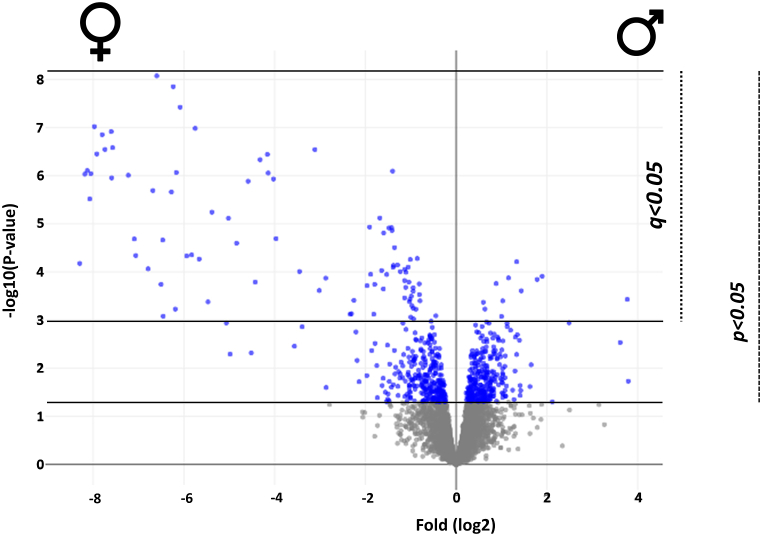
Volcano plot depicting protein with different abundances in the eye of male and female zebrafish created by OmicsLoupe. The protein abundance ratio of male/female was plotted as log2 fold change against the -log10 (*p*-value) of the LIMMA *t*-test, and the Benjamini-Hochberg adjusted p-value was used to set the q-value/FDR 0.05 limit.

**Fig. 2:**
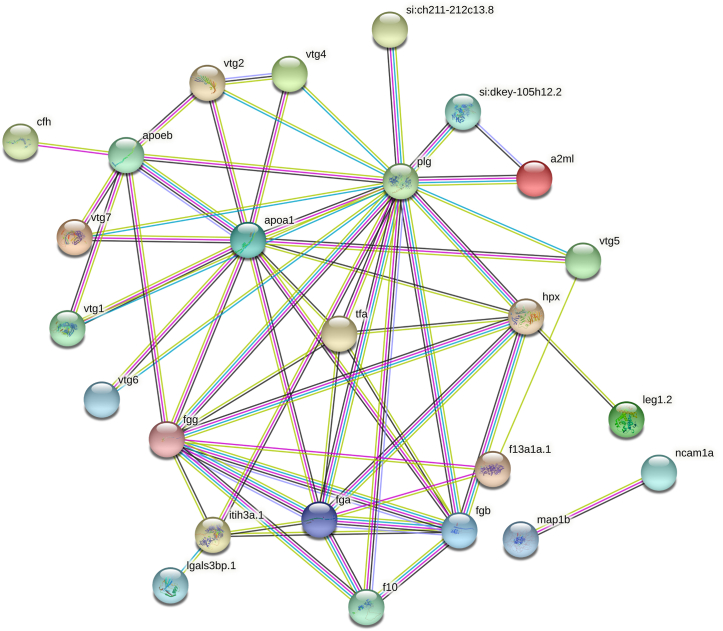
Network map shows the interaction among significantly different proteins between male and female zebrafish eye created using STRING (v11.0). Interactions are shown for text mining, experiments, curated database, co-expression and co-occurrence.

Proteomics analysis identified and quantified 5046 protein group entries in the zebrafish eye samples. After removing 14 decoy (reverse) and redundant entries, 3740 unique protein groups were retained at an estimated FDR (at the protein group level) clearly below 1 % (Supplementary Table S1, Supplementary Fig. S1). Five-hundred and forty proteins, of which 275 and 265 in females and males, respectively, showed higher abundances in comparison with the opposite sex (p < 0.05). After multiple hypothesis correction (q < 0.05), 49 proteins, including 39 and 10 proteins in females and males, respectively (Table 1, Fig. 1), showed higher quantities compared to the other sex, and these were mainly used for the discussion in the present study. Fig. 2 shows interactions among the proteins that are significantly different between male and female zebrafish eyes. Supplementary Table S2 contains detailed information regarding protein interactions, including experimental, database, text mining, related references, cooccurrence, and coexpression evidence that was generated by STRING. To set the protein level differences into context, these proteins were grouped into functional groups and discussed further in the context of existing research.

**Table 1:** Proteins significantly different in the eye of male and female zebrafish. F = female, M = male.

Accession number	Protein name	Gene name	Trend	Function (Uniprot.com)
A0A0G2KTK1	VWFD domain-containing protein	Unknown, vtg2	F > M	Lipid transport, response to estrogen and estradiol, phagocytosis
A0A0G2L365	Myosin-7	LOC100329813	M > F	Cytoskeleton
A0A0R4IAD9	Solute carrier family 38 member 3 b	Slc38a3b	M > F	Amino acid transmembrane transport
A0A0R4IAW3	Neural cell adhesion molecule 1a	Ncam1a	M > F	Cell adhesion, posterior commissure morphogenesis
A0A0R4IDD1	Alpha-2-macroglobulin-like	A2ml	F > M	Serine-type endopeptidase inhibitor
A0A0R4IHQ0	Serotransferrin	Tfa	F > M	Iron hemostasis, antibacterial humoral response
A0A0R4IIB1	Complement factor H	Cfh	F > M	Regulation of complement-dependent cytotoxicity
A0A0R4IJF0	Fibrinogen gamma chain	Fgg	F > M	Blood coagulation
A0A0R4IRJ8	Chloride intracellular channel protein	Clic1	F > M	Chloride transport, glutathione metabolic process
A0A2R8PZ72	Vitellogenin 5	Vtg5	F > M	Lipid transport, nutrient reservoir, response to estrogen and estradiol
A0A2R8Q212	Vitellogenin 1	Vtg1	F > M	Lipid transport, nutrient reservoir, response to estrogen and estradiol, antioxidant, response to xenobiotic
A0A2R8Q549	Vitellogenin 2	Vtg2	F > M	Lipid transport, nutrient reservoir, response to estrogen and estradiol, phagocytosis
A0A2R8Q5K5	Vitellogenin 5	Vtg5	F > M	Lipid transport, nutrient reservoir, response to estrogen and estradiol
A0A2R8Q6H5	2,3-bisphosphoglycerate 3-phosphatase	Minpp1a	F > M	Acid phosphatase activity, bisphosphoglycerate 3-phosphatase activity
A0A2R8QBG3	Vitellogenin 6	Vtg6	F > M	Lipid transport, nutrient reservoir, response to estrogen and estradiol
A0A2R8QEH1	Vitellogenin 1	Vtg1	F > M	Lipid transport, nutrient reservoir, response to estrogen and estradiol, antioxidant, response to xenobiotic
A0A2R8QN47	Vitellogenin 4	Vtg4	F > M	Lipid transport, nutrient reservoir, response to estrogen and estradiol
A0A2R8QNH7	Vitellogenin 7	Vtg7	F > M	Lipid transport, nutrient reservoir, response to estrogen and estradiol
A0JMK3	Zgc:153913	Zgc:153913	F > M	Enzyme regulator activity, protein stabilization
A2BHN0	Annexin	Anxa3a	F > M	Calcium binding, phospholipid binding, phospholipase inhibitor
A5PF59	Liver-enriched gene 1, tandem duplicate 2	Leg1.2	M > F	Liver and digestive system development
B0UXN0	Sulfhydryl oxidase	Qsox1	F > M	flavin-linked sulfhydryl oxidase, protein disulfide isomerase, protein folding
B2ZZ14	Hypothetical microtubule-associated protein 1 b	Map1b	M > F	Actin and microtubule binding, neurogenesis
B7ZD02	Erlin-1	Erlin1	F > M	Ubiquitin protein ligase binding, steroid metabolic process
B8A5L6	Fibrinogen alpha chain	Fga	F > M	Blood coagulation
E7F381	WD repeat domain 26a	Wdr26a	F > M	Proteasome-mediated ubiquitin-dependent protein catabolic process
E7F8T4	Sb:cb37	Sb:cb37	F > M	Serine-type endopeptidase inhibitor activity
E7F9R1	Sodium/potassium-transporting ATPase subunit beta	Atp1b4	M > F	Sodium and potassium transport
E9QBB8	Apolipoprotein Eb	Apoeb	F > M	Lipid transport, lipoprotein metabolic process
E9QFG3	Solute carrier family 24 member 2	Slc24a2	M > F	calcium, potassium: sodium antiporter, long-term synaptic depression, and potentiation
E9QGF2	Plasminogen	Plg	F > M	Endopeptidase, proteolysis, anti-coagulation
F1Q6K5	Lectin, galactoside-binding, soluble, 3 binding protein b	Lgals3bp.1	F > M	scavenger receptor activity
F1Q890	Plasminogen	Plg	F > M	Endopeptidase, proteolysis, anti-coagulation, tissue remodeling
F1QC84	Coagulation factor XIII, A1 polypeptide a, tandem duplicate 1	F13a1a.1	F > M	Blood coagulation
F1QLC3	Coagulation factor X	F10	F > M	Blood coagulation
F1QTF9	Inter-alpha-trypsin inhibitor heavy chain 3a	Itih3a.1	F > M	Serine-type endopeptidase inhibitor, hyaluronan metabolic process
O42363	Apolipoprotein A-I	Apoa1	M > F	HDL particle assembly, cholesterol, and phospholipid efflux
O42364	Apolipoprotein Eb	Apoeb	F > M	HDL particle assembly, lipid homeostasis, negative regulation of neuron apoptotic process, positive regulation of nitric-oxide synthesis
Q1L8D8	Si:rp71-15k1.1	Si:rp71-15k1.1	F > M	Serine-type endopeptidase, protein quality control
Q1LWJ1	Purine nucleoside phosphorylase	Pnp6	F > M	Nucleoside metabolic process
Q1LXK0	Keratin, type 1, gene 19 d	Krt1-19 d	F > M	Cytoskeleton
Q5RHE5	Si:dkey-90m5.4	Si:dkey-90m5.4	F > M	Phospholipase A2 inhibitor, brown fat cell differentiation, positive regulation of angiogenesis, endothelial cell proliferation, transforming growth factor beta receptor signaling pathway, response to bacterium
Q6NYE1	Fibrinogen beta chain	Fgb	F > M	Blood coagulation
Q6PHG2	Hemopexin	Hpx	M > F	Iron homeostasis, response to estrogen stimulus, heme metabolism
Q8AYM7	Green-sensitive opsin-3	Opn1mw3	M > F	Phototransduction, visual perception
X1WBT0	Si:dkey-105h12.2	Si:dkey-105h12.2	F > M	Serine-type endopeptidase inhibitor
X1WC44	Si:ch211-212c13.8	Si:ch211-212c13.8	F > M	Serine-type endopeptidase inhibitor
X1WE71	Transferrin-a (Fragment)	Tfa	F > M	Antibacterial humoral response, hemoglobin biosynthetic process, iron transport
X1WFR7	Collagen, type IV, alpha 4	Col4a4	F > M	Cytoskeleton

### 3.1 Lipoproteins

**Fig. 3:**
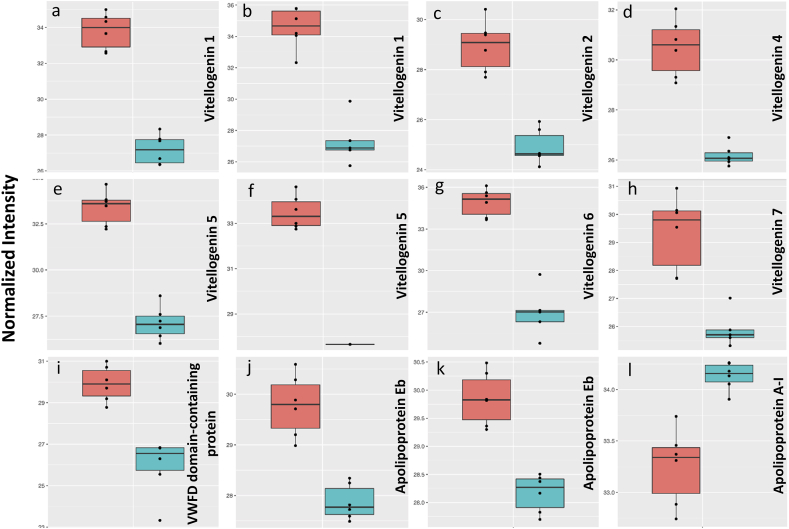
Normalized abundances (log2) of lipoproteins significantly different in the eye of male and female zebrafish. a, b: Vtg1 (A0A2R8Q212, A0A2R8QEH1, respectively), c: Vtg2, d: Vtg4, e, f: Vtg5 (A0A2R8PZ72, A0A2R8Q5K5, respectively), g: Vtg6, h: Vtg7, i: VWFD Domain-Containing Protein, j, k: Apoeb (Q42364, E9QBB8, respectively), l: Apoa1. In each chart, left and right boxes represent females and males, respectively.

Higher levels of vitellogenins (phosvitins, Vtgs) were observed in female zebrafish eye proteome (Fig. 3 a-i). Female zebrafish actively reproduce large numbers of eggs to create new generations, and Vtgs are the main precursor of egg yolk [24] and a carrier of cholesterol produced in the liver and delivered into ovaries via the cardiovascular system. Therefore, at first glance, it is reasonable to have higher levels of Vtgs in females for reproductive purposes. It is assumed that such materials may affect the non-sexual tissues and organs by the extra pressures they impose during their transport to ovaries [1]. The amino acid sequences of Vtgs are similar to apolipoprotein B in human, which is a main component of low-density lipoprotein (LDL) [25]. It is believed that high levels of such lipoproteins and their oxidations are deleterious for eye health [26]. Also, they may have other functions in non-sexual organs beyond their reproductive purposes, such as limb regeneration [27]. Fluctuations in Vtgs levels were observed during the degeneration and regeneration phases of the zebrafish retina [11]. The potential effects of such sex-based protein level differences on the regeneration ability of the retina would be interesting to follow up in future studies.

Apolipoprotein Eb (Apoeb) and apolipoprotein A-I (Apoa1) were significantly higher in the eyes of female and male zebrafish, respectively (Fig. 3j-l). They are important components of lipoproteins and participate in cholesterol homeostasis [28,29]. While the quantity of Apoeb is reported to be similarly higher in the female zebrafish blood in the main vessels and heart, there is no such report for higher Apoa1 in males outside of the eye at the protein level [1,30]. However, it has been shown that the level of Apoa1 is higher in the liver of male zebrafish at transcript level [31].

The gene polymorphism of Apoe is considered a risk factor for age-related macular degeneration (AMD). In addition, Apoe was detected to be deposited in the lesions caused by AMD [32,33]. Also, it has been shown that elevated levels of Apoe and Apoa1 can increase the high-density lipoprotein (HDL) cholesterol associated with a higher risk of AMD [28,29]. However, most of the literature emphasized the role of Apoe in AMD development rather than Apoa1. High levels of lipids transportation via the cardiovascular system into ovaries for oogenesis or embryonic development in females triggers increased levels of Apoeb and subsequently HDL to protect cardiovascular system [1], which on the other hand, is considered a risk factor for AMD. Obesity is a risk factor for eye health [34]. A higher prevalence of obesity was observed among women that was partially caused by their reproductive role [35,36]. This could be considered a bigger risk factor for female eye health and one of the reasons for their higher incidence of visual disorders. Further research can clarify the potential effects of sex-based differences in the quantities of apolipoproteins on male and female eye function and health.

### 3.2 Immune system

**Fig. 4:**
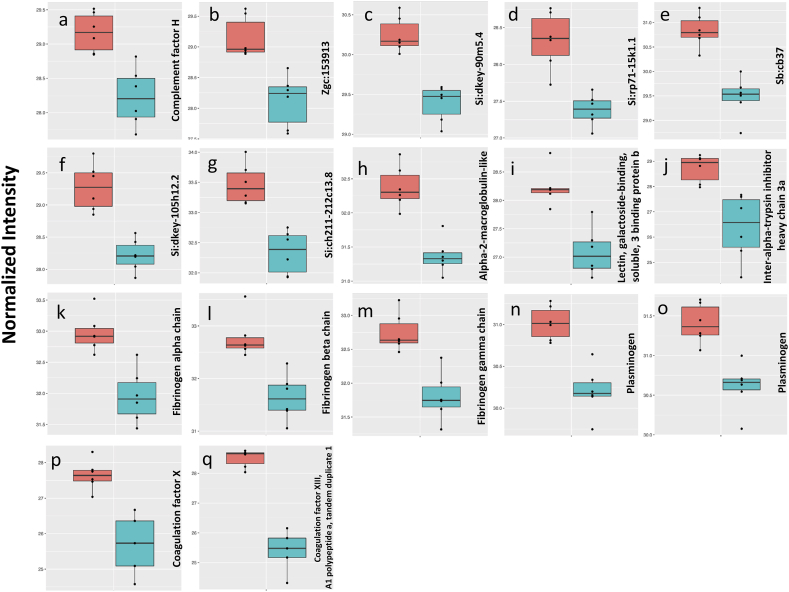
Normalized abundances (log2) of immune system and coagulation system proteins significantly different in the eye of male and female zebrafish. a: Cfh, b: Zgc:153913, c: Si:Dkey-90m5.4, d: Si:Rp71-15k1.1, e: Sb:Cb37, f: Si:Dkey-105h12.2, g: Si:Ch211-212c13.8, h: A2ml, i: Lgals3bp.1, j: Itih3a.1, k: Fga, l: Fgb, m: Fgg, n, o: Plg (E9QGF2, F1Q890, respectively), p: F10, q: F13a1a.1. In each chart, left and right boxes represent females and males, respectively.

We observed a higher level of complement factor H (Cfh) in the eye of female zebrafish (Fig. 4a). A similar female-biased trend for Cfh was observed in zebrafish heart-associated proteins [1]. This protein is a major regulator of the alternative complement pathway, and its dysregulation is a key step for the development of AMD [37]. It was shown that retinal pigment epithelium can synthesize Cfh. Also, Cfh was detected to be accumulated in the lesions of AMD [38,39]. The Cfh gene polymorphism in humans can be either a risk for or protection against AMD development via the change in its expression level and binding efficiency [38,40]. The normal human variant of Cfh protects the eye by competing with lipoproteins for binding and even removing them from AMD development sites. On the other hand, the competition for binding is reduced in Cfh AMD-risk variant which can lead to a faster accumulation of lipoproteins and earlier development of AMD [37,40]. In addition, aging can weaken the immune system and reduce normal variant Cfh level, and its protective effect against lipoproteins that is considered a risk factor for AMD especially when high level of lipoprotein and subsequent obesity retained at the older age [37]. It is an important issue for women since the prevalence of obesity is higher among them [35], which may partially explain their higher rate of AMD. Therefore, a higher level of Cfh in the female zebrafish eye could be attributed to its protective functions against lipoprotein accumulation [37] and oxidative stress [41] caused by the high level of lipoproteins observed in this sex. If the same trend of female-biased Cfh level is true for mammals, the potential effects of higher quantities of this protein's variants on protection from/pathogenesis of AMD in females compared to males, would be interesting to investigate.

We detected a higher level of Zgc:153913 in the eye of female zebrafish (Fig. 4b). The BLAST search found carboxypeptidase N (Cpn) in mouse and human as a similar protein. The inactivation of some immune-related proteins, such as complement anaphylatoxins is the most important function of Cpn that protects the body against excessive activation of the immune system [42,43]. It seems that higher levels of such proteins are necessary since the female has higher quantities of immune proteins and, therefore, a stronger immune system. For example, we found three immune system-associated proteins, namely pentaxin (Crp2), complement component 7 b (C7b), and microfibril-associated glycoprotein 4-like (Loc100334800) only in the female zebrafish eye (Supplementary Table S1). Also, higher quantity of Si:dkey-90m5.4 was found in the female zebrafish eye (Fig. 4c) which is supposed to participate in neutrophil degranulation pathway [44].

Higher quantities of one protease (Si:rp71-15k1.1) and multiple protease inhibitors (Sb:cb37, Si:dkey-105h12.2, Si:ch211-212c13.8, alpha-2-macroglobulin-like, Fig. 4d–h) were observed in the zebrafish female eye. A similar sexual dimorphism was observed in zebrafish heart-associated proteins [1]. The protease inhibitors protect the body against the excessive reaction of immune and coagulation systems, which are mediated via protease activities [45]. Since levels of immune and coagulation-related proteins are higher in females, it is reasonable to balance protease/protease inhibitor (P/PI) levels to ensure proper body function. For example, an imbalance in their synthesis can lead to the destruction of normal tissue (P > PI) or fibrosis (P < PI) during corneal wound healing [46]. In addition, a higher level of alpha-2-macroglobulin in glaucoma showed to have a neurotoxic effect and caused apoptosis in retinal ganglion cells. Interestingly, when alpha-2-macroglobulin was injected into normal and healthy eyes, the same damages were observed without causing other symptoms of glaucoma such as hypertension [47,48]. Sex-biased levels of such protease inhibitors should be considered when their potential protective/destructive effects are studied in male and female visual health and disorder.

Lectin, galactoside-binding, soluble, 3 binding protein b (Lgals3bp.1), a protein with scavenger receptor activity, was found at higher levels in the female eyes (Fig. 4i). Such proteins are on the surface of immune cells to help clearing oxidized lipoproteins, bacteria, and apoptotic cells. Female zebrafish has to transport large quantities of lipoproteins in the vessels toward the ovary that needs higher amounts of such scavenger receptors to reduce the risk of deposition of oxidized lipoproteins in vessel walls [1]. The same process is expected for the protection of female's eye. It was shown that the deposition of oxidized lipoproteins can initiate early events of AMD in the retina [26].

A higher level of inter-alpha-trypsin inhibitor heavy chain 3a (Itih3a.1) was detected in the female zebrafish eye (Fig. 4j). A similar trend was observed in the heart of zebrafish and attributed to its ability to control the potential negative effects of inflammatory proteins, which were detected in higher quantities in female's heart [1]. Also, Itih3a.1 is a major component for extracellular matrix stabilization. Any change in the extracellular matrix can affect cell development and functions by regulating its abilities for differentiation, attachment, movement, and interaction with other cells [49,50].

### 3.3 Blood coagulation

Several proteins involved in the regulation of the coagulation process, including fibrinogen alpha (Fga), beta (Fgb) and gamma (Fgg) chains, plasminogen (Plg), coagulation factors X (F10) and XIII, A1 polypeptide a, tandem duplicate 1 (F13a1a.1) showed higher quantities in female zebrafish eye (Fig. 4 k-q). Similar sexual dimorphisms of coagulation protein levels were found in other limbs of zebrafish [1,30]. It is known that there is crosstalk and interplay between immune and coagulation systems, and each can modulate the other system's activities [51]. Since a stronger immune system exists in females, the same situation can be expected in the coagulation system. Some proteins involved in blood coagulation, such as fibrinogen and coagulation factor X, were detected as the constituents of the lesion created in AMD [52,53]. Also, the immune system plays an important role in the development of AMD [37,54]. Therefore, it is important to investigate the potential effects of sex-biased levels of coagulation proteins and the interplay between immune and coagulation systems on the development of ophthalmic disorders in each sex.

### 3.4 Antioxidants

**Fig. 5:**
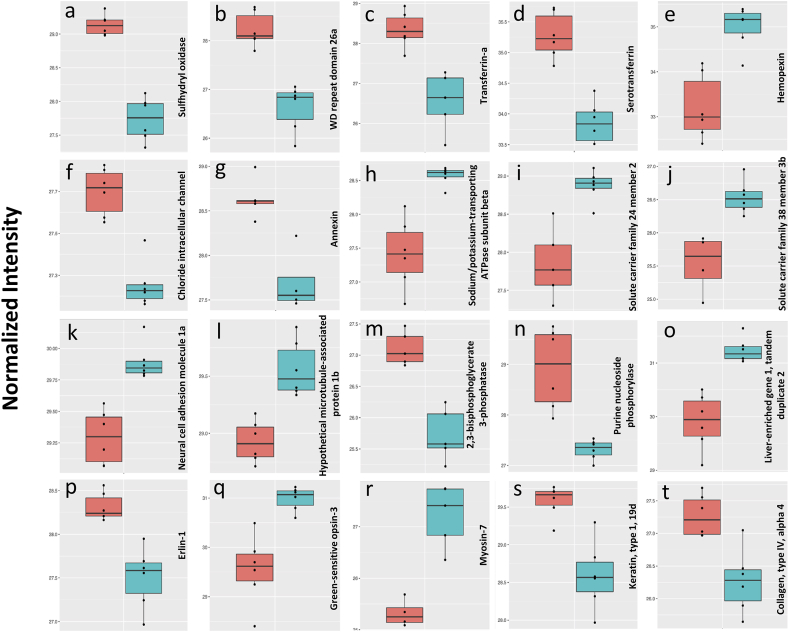
Normalized abundances (log2) of antioxidant, iron and heme binding, ion channels, pumps, and exchangers, neuronal and photoreceptor and cytoskeleton proteins significantly different in the eye of male and female zebrafish. a: Qsox1, b: Wdr26a, c, d: Tfa (X1WE71, A0A0R4IHQ0, respectively), e: Hpx, f: Clic1, g: Anxa3a, h: Atp1b4, i: Slc24a2, j: Slc38a3b, k: Ncam1a, l: Map1b, m: Minpp1a, n: Pnp6, o: Leg1.2, p: Erlin1, q: Opn1mw3, r: LOC100329813, s: Krt1-19 d, t: Col4a4. In each chart, left and right boxes represent females and males, respectively.

A higher level of sulfhydryl oxidase (Qsox1) was detected in the female zebrafish eye (Fig. 5a). This protein protects cardiac cells during stress by coping with oxidative stress and inflammation, proper protein folding, and calcium handling [55]. Therefore, female-biased Qsox1 in the heart of zebrafish was attributed to higher stress that females must endure as a result of the huge quantity of lipids and other materials which are carried in the cardiovascular system [1]. A similar reason could be envisaged for the female-biased Qsox1 in the eye. In addition, Qsox1 likely participates in extracellular matrix formation, and its expression is highly correlated with proteins such as collagen IV [56], which is another protein showing a sex-biased pattern in the zebrafish eye. Also, this protein affects neuron development [56], and it is interesting to investigate its potential effects on optic nerves of different sexes.

WD repeat domain 26a (Wdr26a*)* is a member of wd-40 proteins specialized for various functions such as regulation of cell cycle, intracellular signal transduction, apoptosis, etc. It is observed that the level of Wdr26 is upregulated in response to oxidative stress and protects neural cells against cell death [57]. Large amounts of materials that are transported in the vessels of female zebrafish for reproductive purposes are potential sources of higher oxidative stress across the body of this sex [1], which could be a potential reason for the higher quantity of Wdr26a in females in the present study (Fig. 5b). Sex-biased levels of proteins such as Minpp1, annexin, and Wdr26a may indicate higher levels of stress and susceptibility to apoptosis in female eyes.

### 3.5 Iron and heme binding proteins

While higher quantities of transferrin-a and serotransferrin (Tfa) were found in the female eye, the level of hemopexin (Hpx) was higher in the eye of male zebrafish (Fig. 5c–e). Similar trends were observed in the zebrafish heart-associated proteins [1]. The binding of Tfa can reduce the oxidative stress caused by the toxic free form of iron [58,59] and deprive pathogens of iron that they need for propagation [60]. Also, it was speculated that the level of Hpx, a heme-binding protein, is downregulated in females, perhaps because its anti-inflammatory trait [1,61]. Iron is a necessary element in the retinoid visual cycle. However, a high level of free iron is toxic. Also, iron can be accumulated during aging in retina and cause degeneration [59]. The Tfa can bind free iron but not to the iron in the structure of free heme. Heme is released from damaged erythrocytes and can cause oxidative stress. It was shown that Hpx is produced by neural retina cells, binds heme, and protects the retina against its oxidative stress [58]. Transferrin is synthesized in retinal pigment epithelial cell [62] and protects retina against iron toxicity during diseases [63,64]. These proteomic differences in the levels of Tfa and Hpx may reflect sex-biased approaches for handling iron in the eyes of each sex, which could affect the health and function of their visual system in different ways. Also, while a significantly higher level of transferrin receptor was found in female zebrafish heart-associated proteomic profile [1], such difference was not found in the eye. The transferrin receptor imports transferrin-bound iron into the cell [65]. It may indicate that the level of iron uptake by cells is regulated in sex- and tissue-specific manners.

### 3.6 Ion channels, pumps, and exchangers

Higher quantities of ion channels, such as chloride intracellular channel protein (Clic1) and annexin (Anxa3a) were detected in the female zebrafish eye. On the other hand, significantly higher levels of ion pumps and exchangers such as sodium/potassium-transporting ATPase subunit beta (Atp1b4) and solute carrier family 24 member 2 (Slc24a2) were observed in the male eye (Fig. 5f–j). The chloride channels participate in lens volume regulation [66] and may play a role in corneal wound healing [67]. It is also stated that the intracellular chloride channel is important for the survival and function of the retina and optic nerve in zebrafish and human [68]. The annexins function as a calcium channel that participates in coagulation, inflammation, stress, apoptosis, and phospholipase inhibition [69].

Sodium/potassium-transporting ATPase was detected in the cornea, lens, retina, and related optic nerve [[70], [71], [72]]. It is believed that such proteins have crucial roles in generating and transmitting neuron action potential or nerve impulse [72]. It maintains corneal transparency by regulating its hydration and thickness [70]. Also, this protein is responsible for lens ion homeostasis by active extrusion of sodium to avoid lens cataract and swelling [73]. It has been proven that its activity is inhibited during lens cataract [74].

The solute carrier family 24 member 2 is a potassium-dependent sodium-calcium exchanger. Such exchangers exist in the lens and retina's photoreceptors [75]. They participate in the sensory functions of rod and cone photoreceptors that mediate night-time dim-light vision and daytime colour vision in the retina, respectively [76,77]. Differences in such proteins may affect visual perception in different sexes. Besides sodium pumps, calcium exchangers and pumps play an important role in protecting against cataract by transporting calcium out of the lens. Inhibition of calcium exchanger in the lens caused development of cataract [74,78].

It has been shown that removing the protective effects of estrogen after menopause could be a cause for a higher rate of cataract in females [4]. Based on our results, lower quantities of ion pumps and exchangers in the female eye and subsequently lower capacity of the female eye for regulation of ions could be another reason for a higher cataract rate in females, especially at older ages when protective effects of estrogen do not exist. The cortical cataract is dominant in female [4] in which changes in ion concentrations such as calcium, sodium, potassium, etc., were observed [73] that may strengthen the hypothesis for a lower ion regulatory ability in the female eye during cataract development at older ages.

Also, it is interesting to address the question of whether different abilities of calcium regulation in male and female eyes can affect the dynamics of calcium spherule formation during the development of AMD [39].

A higher quantity of solute carrier family 38 member 3 b (Slc38a3b) was detected in the male zebrafish eye (Fig. 5j). This is a sodium-coupled neutral amino acid transporter that belongs to system N transporters. A study detected this protein in photoreceptors, the inner nuclear layer, glial cells, and the ganglion cells of the retina. This protein participates in the glutamate-glutamine cycle, which is necessary for the synaptic transmission of nerve impulses generated in eye [79].

Interestingly, we only detected sideroflexin-4, a low abundant protein based on the signal intensity, in the eye of males (Supplementary Table S1). This protein may participate in transmembrane transport of ions and amino acids [80], which could be considered another evidence for differences in cellular transport capacity between sexes.

### 3.7 Neuronal and photoreceptor proteins

Higher quantities of neural cell adhesion molecule 1a (Ncam1a) and hypothetical microtubule-associated protein 1 b (Map1b) were found in the eye of male zebrafish (Fig. 5k and l). Both proteins have been shown to have roles in neural development and function [81,82]. Kustermann and colleagues revealed that polysialylated neural cell adhesion molecule participates in the generation of rod photoreceptors in zebrafish retina [83]. Interestingly, Dnajc6, a clathrin-binding protein that may participate in the synaptic transmission via synaptic vesicle uncoating, was only detected in the eye of male zebrafish [80] (Supplementary Table S1). A morphological study of the human retina showed differences in the thickness of macular layers between the two sexes [84]. Significant differences in the quantities of several neuron-associated proteins between the eye of male and female zebrafish may further indicate such differences in retinal neural networks that are reflected at a molecular level.

A higher level of 2,3-bisphosphoglycerate 3-phosphatase (Minpp1a) was recorded in the female zebrafish eye (Fig. 5m). This protein controls inositol polyphosphates which are responsible for the regulation of cell differentiation, vesicular trafficking, calcium mobilization, apoptosis, etc. Without the function of this protein, neurons are not sufficiently differentiated, and their cell death increases. Also, the loss of function of this protein increases the chelation of intracellular iron and calcium, which reduces their free forms in neurons. Also, cataract was observed in the eyes of some patients who lacked such gene [85]. On the other hand, the expression of Minpp1 in the endoplasmic reticulum increases in response to environmental stresses that probably induces apoptosis [86].

Purine nucleoside phosphorylase (Pnp6) in the mammalian lens may act as an energy-saving agent [87]. It is also released from mammalian astrocytes and microglia cells after an inflammatory stimulation [88]. Such proteins may be important for iridophore pigment cell development in zebrafish eye [89]. Also, this protein plays an important role in the metabolism of purines [88]. The physiological functions of purines in the cornea, trabecular meshwork, lens, Müller cells, astrocytes, retinal neurons, and retinal pigment epithelium were also studied [90]. Considering a broad range of Pnp6 and purine functions in the eye, a higher quantity of this protein in females (Fig. 5n) can potentially induce a wide sexual dimorphism in this organ.

Liver-enriched gene 1 (Leg1.2) protects liver development against stress in zebrafish [91]. A phylogenetic investigation revealed functions such as calcium homeostasis, negative regulation of sequestration of triglyceride, synapse organization, etc. [92]. However, further research is required to clarify the potential reasons for a lower quantity of this protein in female zebrafish eye (Fig. 5o).

Erlin (Erlin1) mediates Inositol 1,4,5-trisphosphate receptors, which in turn are responsible for secretion, apoptosis, calcium channel activity, etc. Mutation of erlin is linked to neurodegenerative disorders [93]. Also, erlin negatively regulates the synthesis of cholesterol when the level of this lipid is high [94]. Cholesterol regulation could probably be a reason for the female-biased trend of this protein, as the cholesterol level is higher in females (Fig. 5p) because of reproductive roles.

Results of the present study showed a higher quantity of a type of green-sensitive opsin protein (Opn1mw3) in males (Fig. 5q). Opsins are photoreceptor proteins in the retina which mediate several visual functions, including colour vision. Sexual dimorphism in the levels of expression of opsin genes was reported in the eyes of guppies. Such differences may affect the visual ability of each sex to detect danger, search for food, and select mates [95].

### 3.8 Cytoskeleton proteins

Several cytoskeleton proteins with sex-biased levels were found in the eyes of zebrafish. For example, myosin-7 and keratin, type 1, 19 d were higher in males and females, respectively. Also, collagen, type IV, alpha 4 (Col4a4) was found at higher levels in females (Fig. 5r-t). Based on their wide range of functions in different compartments, some structural and functional sexual dimorphism can be expected in the eye. However, it is difficult to point out some specific effects of such structural proteins in the formation of sexual dimorphism in the eye.

It must be mentioned that we found a large number of differentially abundant proteins at p < 0.05. To reduce the false discoveries and reach to the most important proteins, we filtered the data stringently to an FDR of <5 % (q < 0.05), and mainly used these data for discussion. However, there are numerous other proteins found at p < 0.05 with potentially important roles in sexual dimorphism that could be interesting for ophthalmologists. Therefore, we uploaded all raw and results files as supplementary data to allow scientists in the field to reuse and explore them based on the newly developed tools to extract further knowledge that might be unseen in the present study.

## 4 Conclusion

The results of the present study showed sex-based differences in the quantities of many zebrafish ocular proteins involved in lipid transport, immune system, antioxidants, blood coagulation, iron and heme-binding, calcium and other ions transport, neuronal transmission, colour vision, and cytoskeleton. The process of sex-biased ocular impairments may be mediated, accelerated, or postponed, at least partly, by the differences in the quantities of some proteins, such as lipoproteins, ion transport, etc., in the eyes of males and females. The differences in the quantities of such proteins in the eye may affect the susceptibility and outcome of visual disorders such as cataract and AMD in females. Such basic knowledge may enhance our understanding towards the development of therapeutic approaches for the improvement of the visual health of the susceptible sex. Ions such as sodium, potassium, calcium, etc., play crucial roles in the photoreceptors and optic nerve for the generation and conduction of nerve impulses and the creation of visual perception. Sex-biased differences in the quantities of ion channels, pumps, and exchangers may affect characteristics of nerve impulse generation and transmission (e.g., speed, capacity, etc.) and subsequently visual perception in different sexes. Finally, unequal quantities of photoreceptor proteins such as opsins may provide different sensitivity levels for colour vision between sexes.

## 5 Limitations of the study

The development of high-resolution mass-spectrometry combined with advances in software for data processing and analyses has enabled quantitative proteomics for the high-throughput screening of thousands of proteins in biological systems. However, this method provides many identified and quantified proteins related to different functional categories and subsequently makes it difficult to deduce all possible implications in a single paper. While several of the discussed findings fit well with observations in other studies, the implications of a large proportion of identified and quantified proteins and the ways they shape sexual dimorphism in the eye remain to be explored and functionally validated in future studies. Functional validation was beyond the scope of the present study, but we hope that our findings can help other researchers to select candidates for functional analysis. We have also been searching for other omics datasets which could help to determine the generalizability of dimorphisms and potential factors behind visual impairments, but without success. The deposition of raw data from the present study as supplementary data and in a public repository may thus help researchers integrate these data with future datasets for validation and for the discovery of new aspects of sexual dimorphism.

## Funding

This research was supported by the Faculty of Engineering at 10.13039/501100003252Lund University, through Proteoforms@LU.

## Data availability

The mass spectrometry proteomics data have been deposited to the ProteomeXchange Consortium via the PRIDE [96] partner repository with the dataset identifier PXD033338. The Supplementary Table 1 contains all data regarding identified and quantified peptides and proteins.

## CRediT authorship contribution statement

**Hamid Niksirat:** Writing – review & editing, Writing – original draft, Investigation, Funding acquisition, Formal analysis, Data curation, Conceptualization. **Valentina Siino:** Writing – review & editing, Methodology, Formal analysis, Data curation. **Christoph Steinbach:** Writing – review & editing, Investigation, Formal analysis. **Fredrik Levander:** Writing – review & editing, Software, Methodology, Funding acquisition, Formal analysis, Data curation, Conceptualization.

## Declaration of competing interest

The authors declare that they have no known competing financial interests or personal relationships that could have appeared to influence the work reported in this paper.
