# Supplementary material for: The quantification of zebrafish ocular-associated proteins provides hints for sex-biased visual impairments and perception
Source: Heliyon. 2024 Jun 13;10(12):e33057. doi: 10.1016/j.heliyon.2024.e33057 (PMC11238053; doi:10.1016/j.heliyon.2024.e33057)
Supplement: Multimedia component 1 [file mmc1.pdf]

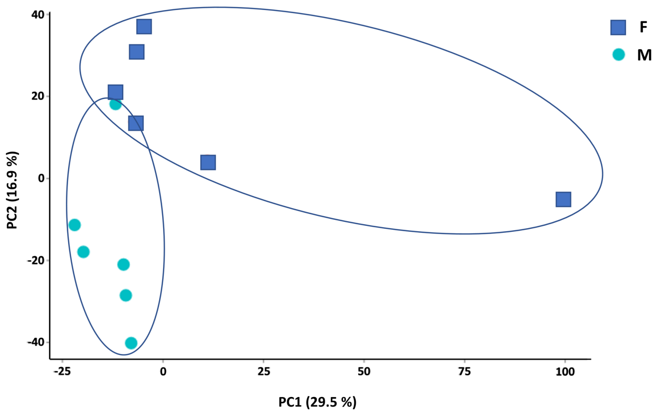

Supplementary Fig.1

Supplementary Fig. 1 First two components of PCA of the eye samples based on quantified protein groups. F=females, M=males.
